# Evaluation of clove-chitosan nanohybrids and clove nanoemulsions as bio-agents against the peach fruit fly, *Bactrocera zonata* (diptera: tephritidae)

**DOI:** 10.1038/s41598-026-57480-8

**Published:** 2026-08-14

**Authors:** Mai K. K. A. Daif, Marwa A. Ramadan, Khaled Yehia Farroh, Amira Afify, Amna H. Faid

**Affiliations:** 1 Plant Protection Research Institute, Dokki, Giza, Egypt; 2 Department of Laser Application in Metrology, Photochemistry and Agriculture, National Institute of Laser Enhanced Science (NILES), Cairo University (CU), Giza, Egypt; 3 Nanotechnology and Advanced Materials Central Lab., Agricultural Research Center, Giza, Egypt; 4 Regional Research Center for Food and Feed, Agricultural Research Center, Giza, Egypt; 5 Biotechnology Department, Faculty of Science, Cairo University, Giza, Egypt; 6 Department of Laser Sciences and Interactions, National Institute of Laser Enhanced Science (NILES), Cairo University, Giza, Egypt

**Keywords:** *Bactrocera zonata*, Biopesticides, Clove, Chitosan, Nano-oil, Nanotechnology, Green synthesis, Biochemistry, Biological techniques, Biotechnology, Nanoscience and technology, Plant sciences, Zoology

## Abstract

Plant Essential oils (EOs)-based eco-friendly biopesticides area substitute for chemically manufactured insecticides in integrated pest management (IPM) programs. Plant essential oils show chemical instability which causes the active ingredients to evaporate and degrade quickly and that is the primary issue for using them as biopesticides in field applications. EOs could be incorporated into controlled-release nano formulations, which are anticipated to be more efficient than the bulk substance. In this research, clove nanoemulsion (CEO-NE) and clove-chitosan nanohybrid (CEO-NE@CNPs) were assessed as bio-agents against the peach fruit fly, *Bactrocera zonata* (Saunders) (Diptera: Tephritidae) under laboratory conditions. Results reveal that upon encapsulation of CEO-NE on chitosan nanoparticles (CNPs), there was an increase in the particle size from 110 ± 10nm to 170 ± 10nm with high stability 50.9mV and 30.5mV respectively. In addition, CEO-NE@CNPs was the most efficient bioagent against *B. zonata* that recorded the lowest egg hatchability and showing the highest larval mortality. Also, low pupation and low values of adult emergence were obtained. So, the combination of chitosan NPs and clovenanoemulsion as a nanohybrid increased the efficacy of clove oil as an ecofriendly pesticide.

## Introduction

Family Tephritidae (order Diptera) contains several highly invasive and harmful insect pests of crops, whose prevalence continues to increase owing to globalization, international trade, and human movement. The fruit fly *Bactocera zonata* (Saunders) (Diptera: Tephritidae) is one of the most destructive fruit flies affecting fleshy fruits of many species and varieties of horticultural crops such as peach, apricot, mangoes, guava, figs, citrus, and others in several temperate, subtropical and tropical countries all over the world.^1,2^ *Bactrocera zonata* was documented for the first time spent in Egypt in 1998 saw rapid expansion and growth. Present in most Egyptian provinces, leading to great losses in all fruit crops.^3^ The infestation with fruit flies quantitatively and qualitatively reduces the fruit yield. Regrettably, the management of tephritid flies is still heavily dependent on the application of synthetic insecticides, which pose negative impacts on both the environment and human health. Also, other control applications for fruit flies were used such as horticultural and phyto-sanitary practices as well as male annihilation technique and post-harvest treatments.^4^

Lately, studies have explored alternative and more eco-friendly tools for biomedical applications^5–9^ particularly for implementation in integrated pest management (IPM) programs^10–13^. In this context, EOs and their primary constituents offer promising substitutes for chemical insecticides. Essential oil compounds and their derivatives serve as viable alternatives for managing numerous harmful insect populations, and their swift degradation in the environment enhances their selectivity towards beneficial insects. A variety of commercial essential oil products are currently being formulated for diverse applications in both human and animal contexts, particularly in pest management ^14–16^.

Several pesticides and essential oils were used in many formulations of nano-emulsions for controlling many pests ^17–21^. Essential oils of clove (*Syzygium aromaticum*) (Myrtaceae) and other plants were widely used as pesticides for many pests^22–24^. Chitosan and oligochitosan are recognized biological control agents for various plant diseases and numerous insect species ^25,26^.

Moreover, earlier research has emphasized the significance of elucidating whether the observed insecticidal effects of essential oils and their nano-formulations occur via ingestion, contact, or fumigant action. This aspect remains critical to understand the precise mode of action and to compare efficacy with conventional pesticides. ^24,25^

This research focused on investigating the bio-efficacy of nano-clove and clove-chitosan emulsions against the *Bactrocera zonata* (Saunders) (Diptera: Tephritidae) under laboratory conditions.

## Material and methods

Essential oil of Clove [Syzygium aromaticum (Myrtaceae)] were obtained from El-Abgy Factory for Extracting Plant Oils. All the chemical used in our research were with purity with purity 99.99% and purchased from Sigma-Aldrich, U.S.A.

### Preparation of CEO-NE

To prepare the 2% v/v nanoemulsion 2ml of clove oil was added to 97 ml double-distilled water at room temperature and 1 ml of Tween 80 as a surfactant. The resulting crude emulsion was sonicated using a 40W probe sonicator (UP400ST, Hielscher, Germany). The sonication time was fixed for 20 min to minimize CEO-NE droplets.^23,27,28^

### Preparation of CNPs and CEO-NE@CNPs

First CNPs were obtained at room temperature by ionic gelation technique as mentioned in our previous publications, using tripolyphosphate (TPP) with chitosan polymer with medium molecular weight dissolved in 0.6% (w/v) with 1% (v/v) acetic acid solution. The pH of chitosan solution was raised to 4.6–4.8 with 1N NaOH. Chitosan nanoparticles formed spontaneously upon addition of an aqueous TPP solution (0.3%, w/v) to the prepared chitosan solutions under magnetic stirring.^29–33^ Then, 2 ml clove oil was added to CNPs sonicated and 1 ml of Tween 80 using a 40W probe sonicator (UP400ST, Hielscher, Germany). The sonication time was fixed for 20 min to minimize EO-CNPs droplets. The resultant nanoemulsions were stored under laboratory conditions (25°C).^21,34^

### Characterization of CEO-NE and CEO-NE@CNPs

The morphology of the prepared solutions was studied via JEOL (JEM-1400 TEM) transmission electron microscope and TEM lab FA-CURP, Faculty of Agriculture, Cairo University Research Park, Images were taken using an optronics CCD camera model AMT, which has a side mount setup and a 1632 × 1632-pixel format. For acquisition, this camera uses a 1394 fire wire board.^9,31,32^ Extremely diluted solutions were positioned on a copper grid that is covered with amorphous carbon and then detected by TEM. An FT-IR spectrometer (Shimadzu FT-IR 8400) was used to perform infrared measurements in the 500–4500 cm-1 range.^35^ Using a lyophilizer, samples were dried. Using a potassium bromide (KBr) pellet, the infrared spectra of powdered samples were diluted. When 4–8 mg of the dried samples was added to 200 mg of KBr, the sample needed to be well ground to remove scattering inaccuracies.

The DLS with Zeta sizer 300 HAS (Malvern Instruments, Malvern, UK) was used to assess the particle size and surface charges of CEO-NE and CEO-NE@CNPs using photon correlation spectroscopy. A 60-s analysis time was used to calculate the average zeta potential.^5,36^

### Insects and oil providing

The peach fruit fly individuals used in the experiments were taken from a laboratory stock continuously reared in a horticultural entomology laboratory. Research Department, Plant Protection Research Institute, Agricultural Research Centre.^37^

### Treatment of insects with bio-agents

**Fig. 1:**
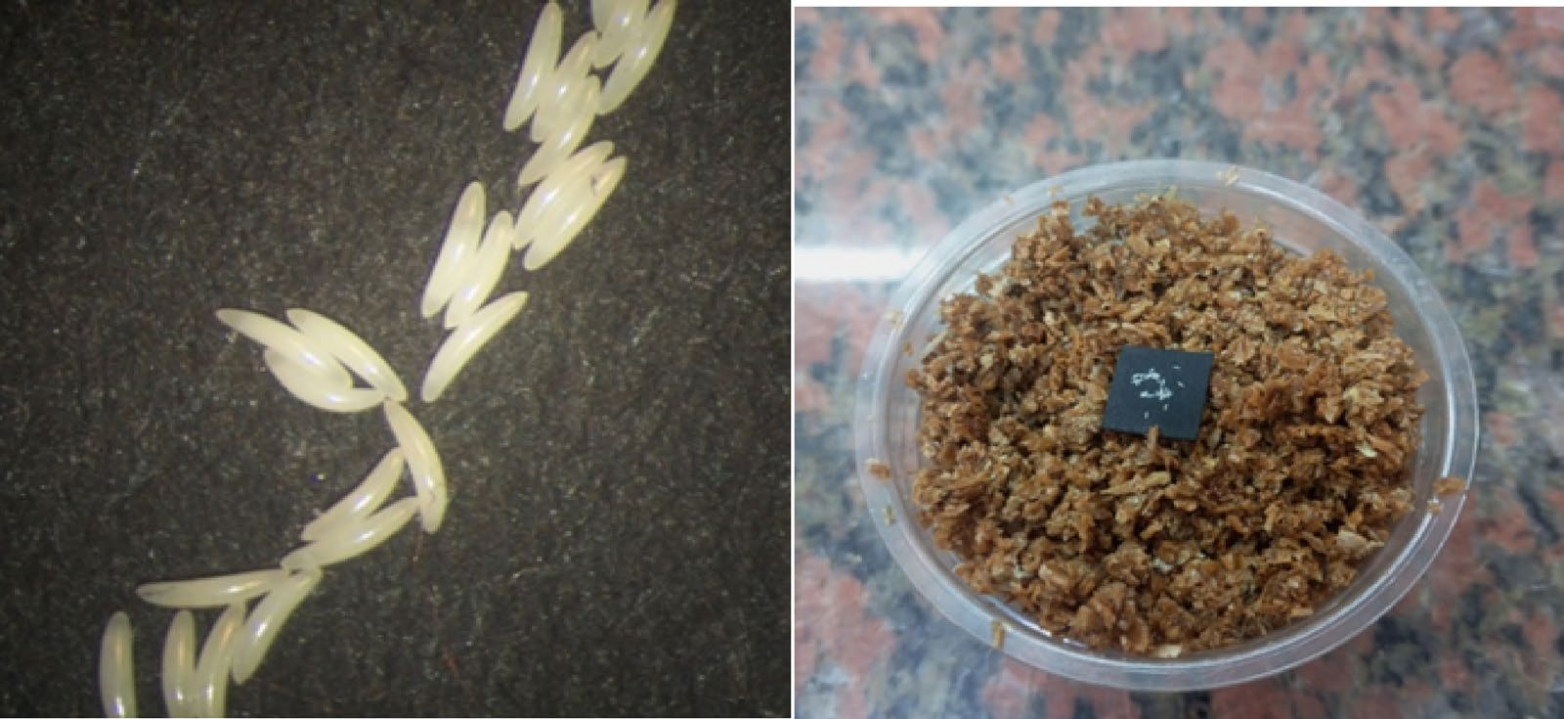
(**A**) Healthy eggs of *B. zonata* and (**B**) Cups of larval artificial diet.

The effects of the above bio-agents, such as gastro-toxins, on the fruit fly at the egg stage and their potential effects at subsequent developmental stages were studied in the laboratory. A concentration of 10% v/w (volume of compounds per weight of diet) was used as shown in Fig. 1.

The various formula quantities were meticulously mixed with the larval diet inside a plastic container. Afterward, each concentration was equally allocated into three replicates within small plastic dishes, where eggs that were 6–15 h old were gently placed on moist black filter paper (1 × 1 cm) on top of the diet. The control group included insects that received no treatment (the eggs were only subjected to the larval diet). Subsequently, the cups were securely covered with muslin fabric fastened in place by rubber bands. The eggs underwent daily evaluations over a span of four days to determine their hatchability. Following this period, they remained undisturbed for seven days until pupation commenced, at which point each cup was transferred to a larger plastic container containing a small amount of sand to aid the larvae’s pupation process. The pupae from each sample were then sifted, counted, and stored in plastic vials until they emerged. All adult insects that emerged were tallied, and any deformed or atypical individuals were noted.^2^

All the data obtained were statistically analyzed according to analysis of variance (ANOVA) (Tukey’s test) with SPSS, and sample significance was determined at P ≤ 0.05. The mean ± standard error of the data (n = 3) is displayed.

## Results and discussion

### Transmission electron microscopy (TEM)

**Fig. 2:**
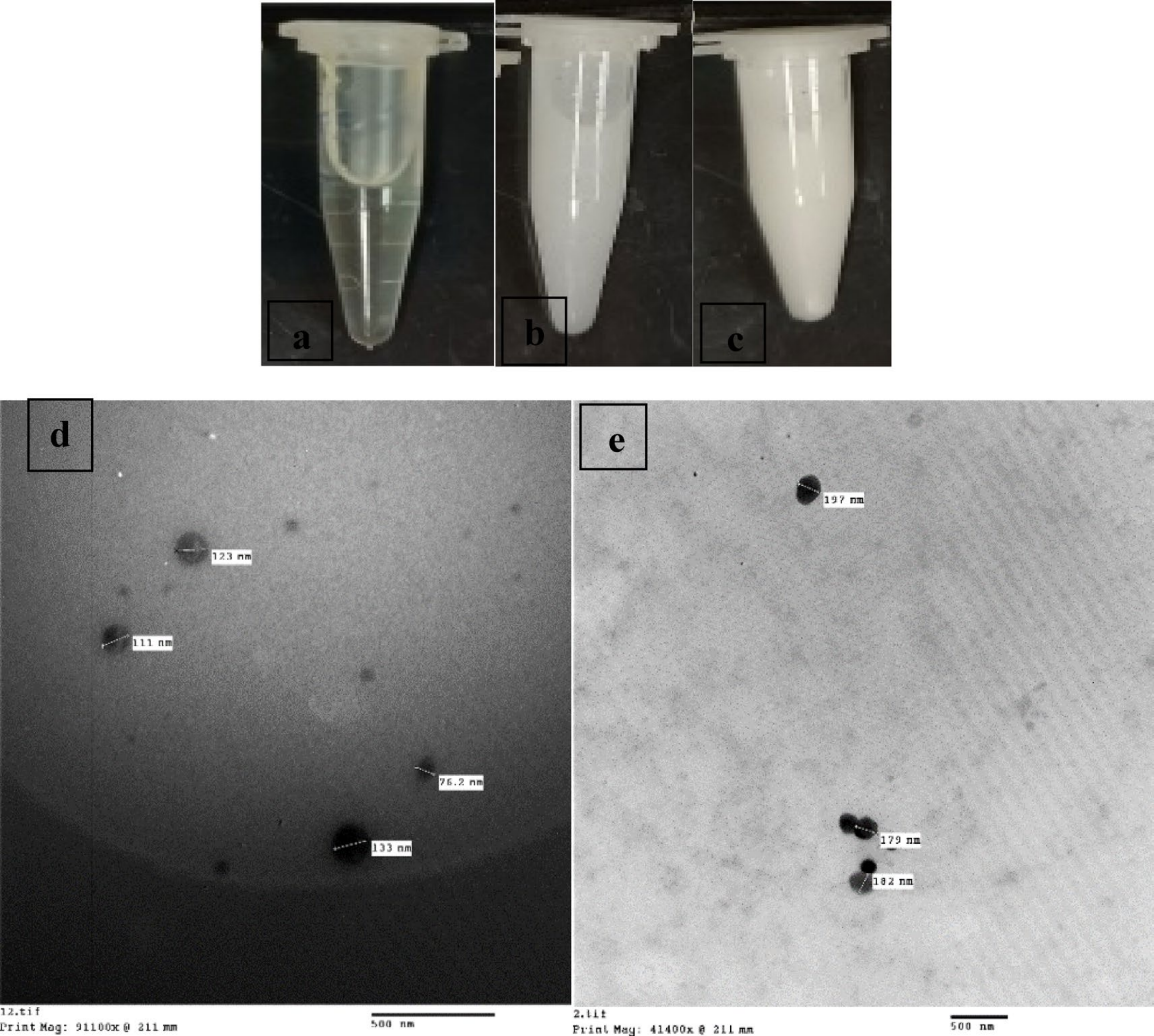
Digital photograph of (**a**) clove oil (CEO) (**b**) clove nanoemulsion (CEO-NE) (**c**) clove-chitosan nanohybrid (CEO-NE@CNPs) and TEM images of (**d**) clove nanoemulsion (CEO-NE) (**e**) clove-chitosan nanohybrid (CEO-NE@CNPs) (magnification 500 nm).

**Fig. 3:**
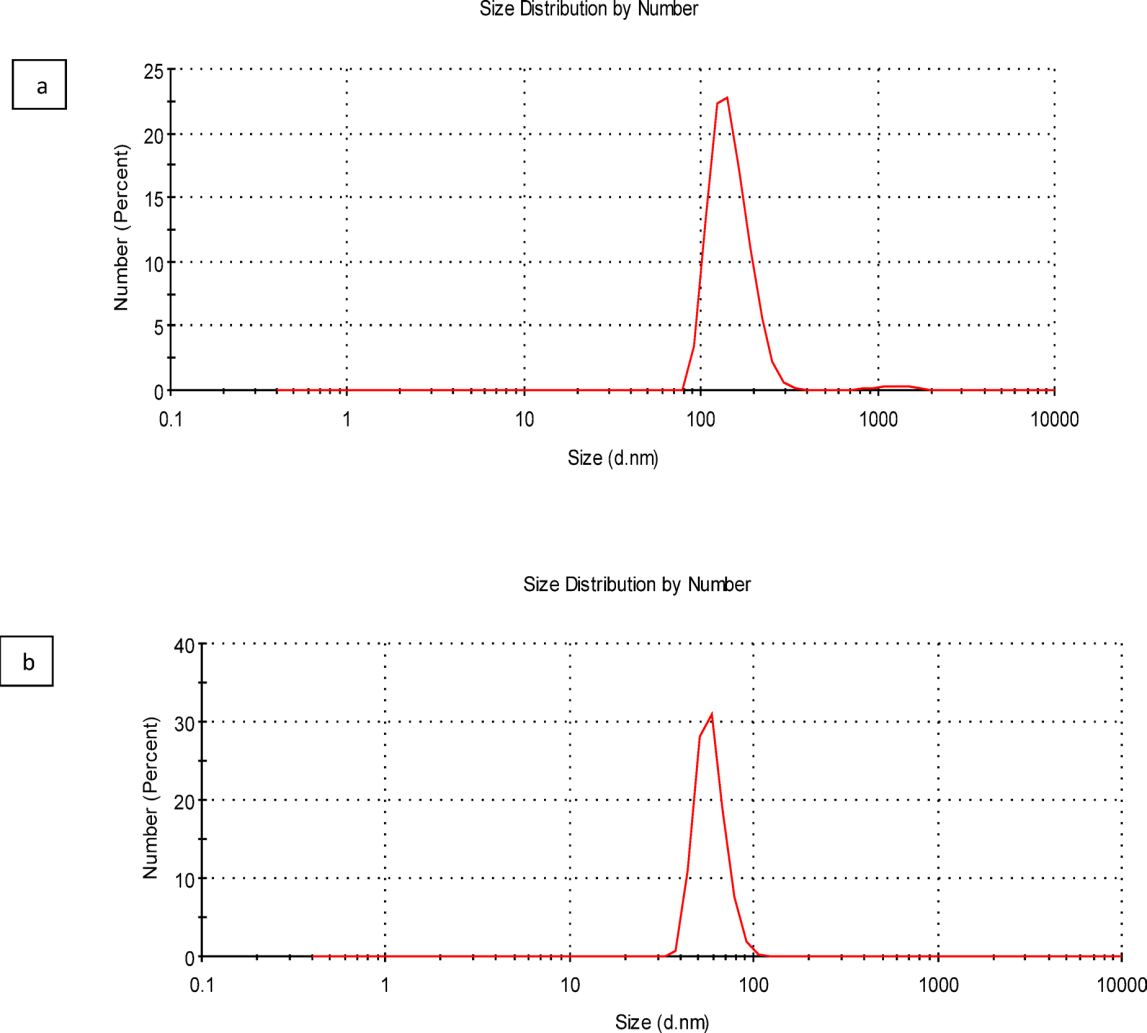
Particle size (**a**) clove nanoemulsion (CEO-NE) (**b**) clove-chitosan nanohyprid (CEO-NE@CNPs).

Figure 2 shows digital photographs of a) clove oil (CEO), b) clove nanoemulsion (CEO-NE) c) clove-chitosan nanohybrid (CEO-NE@CNPs) and TEM images of d) CEO-NE, e) CEO-NE@CNPs. One of the most skilled methods for determining the morphology, structure, and frequency of particle size is TEM examination. CEO-NE droplets are uniform and spherical in Fig. 1a. NE with a spherical shape is known to spread effectively without aggregating^38^. Upon encapsulation of CEO-NE on CNPs Fig. 3b**,** there was an increase in the particle size from 110 ± 10nm to 170 ± 10nm.

#### Size and surface charge of CEO-loaded chitosan NPs:

**Fig. 4:**
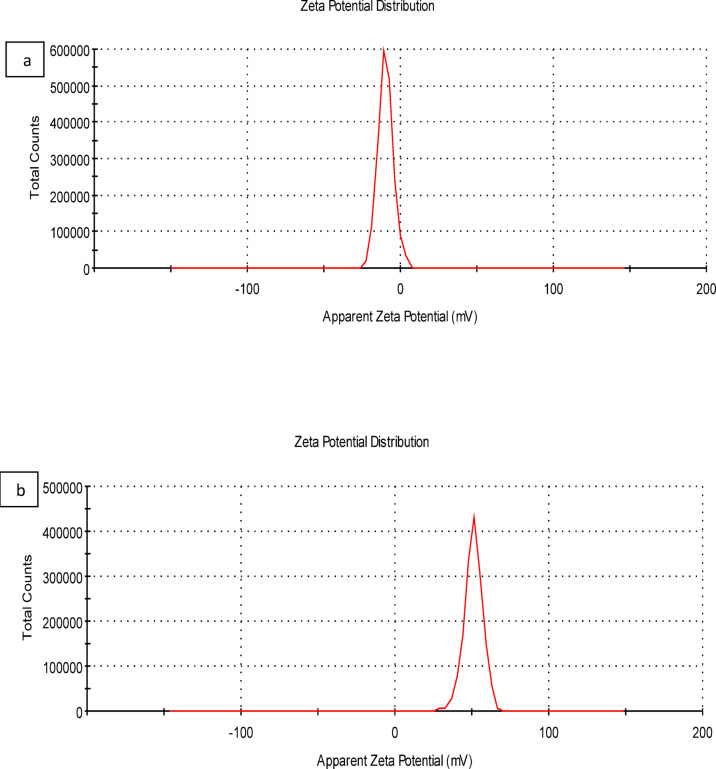
Particle size (**a**) clove nanoemulsion (CEO-NE) (**b**) clove-chitosan nanohyprid (CEO-NE@CNPs).

The results of particle size and zeta potential of clove nanoemulsion (CEO-NE), and clove-chitosan nanohyprid (CEO-NE@CNPs) are presented in Figs. 3, 4. Clove nanoemulsion (CEO-NE) have a mean diameter of 148.2 nm. The size of CNPs was increased by encapsulating CEO-NE to398.2 nm, which accords with the results of Hosseini et al.^39^ detected enlargement of oregano EO-loaded chitosan NPs; they reported that chitosan NPs and oregano EO-loaded chitosan NPs had a particle diameter of 281.5 and309.8 nm, respectively. Zeta potential and Polydispersity Index (PDI) quantify the strength of electrostatic repulsion or attraction between particles, playing a crucial role in processes such as dispersion, flocculation, aggregation, homogeneity and stability. Consequently, it serves as an essential parameter for assessing the stability of dispersions, emulsions, and suspensions. In the present work, zeta potential of clove nanoemulsion (CEO-NE) was found to be -9.6 mV with DPI = 0.677 whereas clove-chitosan nanohybrid (CEO-NE @CNPs) had a positive zeta potential 50.9 mV with DPI = 0. 083.In accordance with our study, Hasani et al. the values of zeta potential for chitosan-Hicapnanocapsules containing lemon EO ranged from + 10.58 to + 44.23 mV. In another research, chitosan NPs and cinnamon EO-loaded chitosan NPs have positively charged surfaces with zeta potential values were + 24.0 mV and + 26 to + 30.5 mV, respectively ^40,41^.

### Chemical structure of CEO-NE -encapsulated chitosan NPs (CEO-NE@CNPs)

**Fig. 5:**
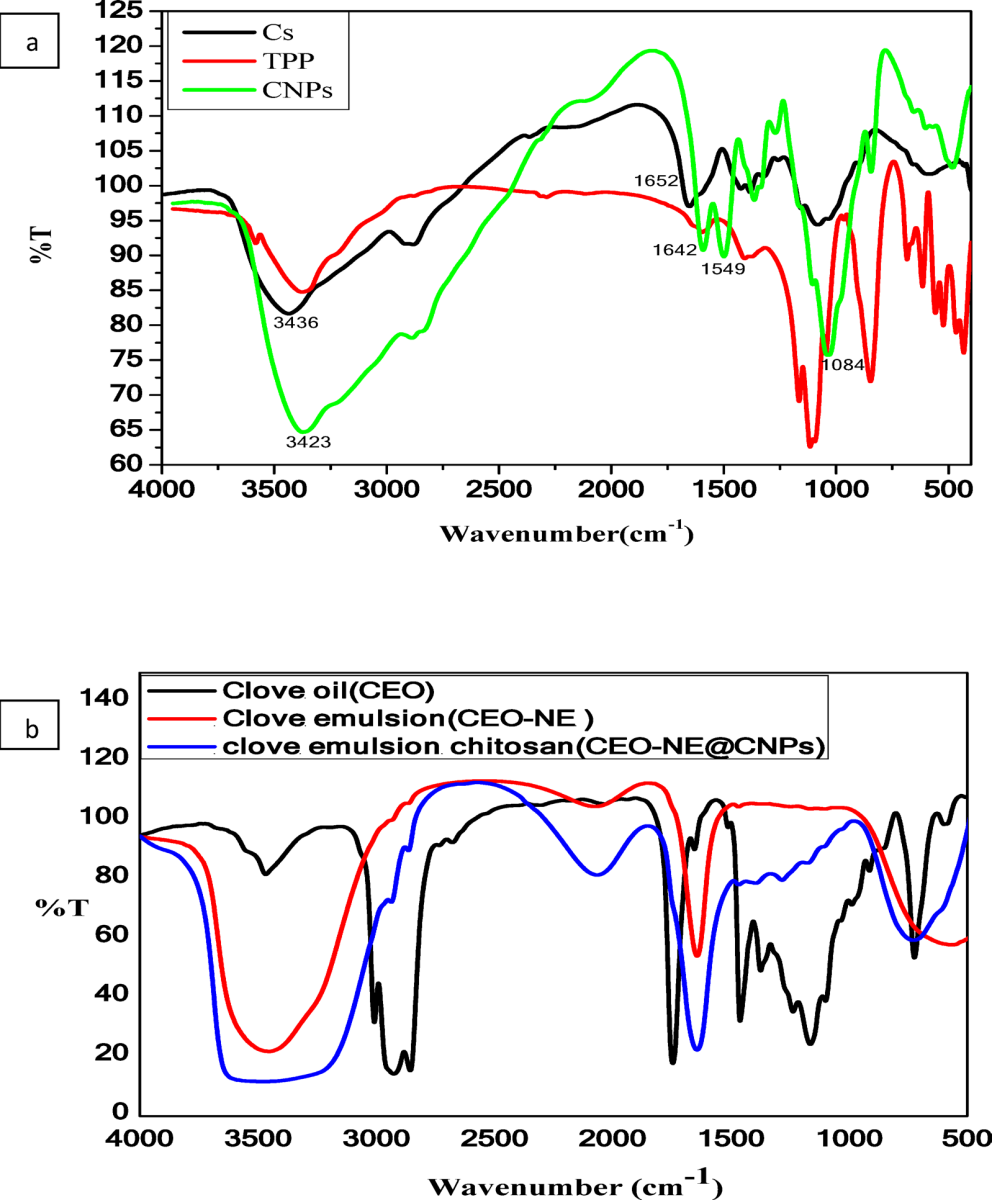
FTIR spectra of (**a**) Cs polymer, TPP, CNPs, (**b**) clove essential oil(CEO), clove nanoemulsion (CEO-NE), clove-chitosan nanohybrid (CEO-NE @CNPs).

FTIR analyses depicted **in **Fig. 5a, for CS, TPP, and CNPs were conducted to elucidate the chemical structure of the CNPs nanoparticles. As illustrated in Fig. 3a, three characteristic peaks of CS are evident; the peak at 3435 cm^−1^ is associated with the hydrogen-bonded O–H stretching vibration, while the peaks corresponding to N–H stretching from primary amine and type II amide overlap in the same region. Additionally, the peak at 1082 cm^−1^ corresponds to ν(C O C), and the peak at 1652 cm^−1^ is associated with the CONH_2_ group^42^. In the TPP spectrum, the following characteristic bands can be identified: 1214 cm^−1^ (P = O stretching), 1148 cm^−1^ (symmetric and antisymmetric stretching vibrations in the PO2 group), 1093 cm^−1^ (symmetric and antisymmetric stretching vibrations in the PO3 group), and 912 cm^−1^ (antisymmetric stretching of the P O P bridge). The spectrum of CNPs differs from that of the chitosan polymer. In CNPs, the peak at 3435 cm^−1^ in the chitosan polymer shifts to 3422 cm^−1^ and broadens with an increase in relative intensity, indicating enhanced hydrogen bonding. The peak at 1652 cm^−1^ in the chitosan polymer shifts to 1642 cm^−1^, with a decrease in intensity, and a new sharp peak at 1549 cm^−1^ corresponding to N–H (Bend) emerges. CNPs also exhibit a P = O peak at 1154 cm^−1^. These findings can be attributed to the linkage between the phosphoric group of TPP and the ammonium group of chitosan in the nanoparticles, which enhances both inter- and intramolecular interactions in CNPs^43^. The terminal phosphate group of TPP forms an ionic bond with the amine NH2 group of chitosan. The ionic interaction with the phosphate group of TPP signifies the transformation of chitosan polymer into CNPs, resulting in a cross-link with TPP. The pronounced and sharp peak of phosphate at 1083 cm^−1^ in CNPs confirms the participation of TPP during the formation of the nanoparticles ^12,44^.

In addition FTIR spectroscopy was used to confirm the interactions between clove essential oil(CEO), clove nanoemulsion (CEO-NE) and clove-chitosan nanohybrid (CEO-NE @CNPs) as shown in Fig. 5b, FTIR spectra of clove oil (CEO)showed peak at3467 cm^−1^ is associated with OH stretching in addition^21^,the peak at 3007 cm^-1^ due was observed due to allyl group (C − H attached to olefin) in eugenol,2925 cm^−1^ and 2855 cm^−1^ are associated with OH and fatty acid stretching, while 1745 cm^−1^ represents the ester C = O group, while the peak at 1373 cm^-1^ indicates the C-H deformation vibration of eugenol methyl. Peaks at 1653 cm^−1^ and 1510 cm^−1^ corresponded to the main peaks due to eugenol. Peaks at 1460 cm^−1^ were due to C–C stretching vibrations of the phenyl ring while the one at 1163 cm^−1^ was due to C–O bending^34^

Furthermore, other vibration modes oil including C-O stretching vibration of carboxylic acid, C = O stretching vibration of phenolic hydroxyl, and the C = C stretching vibration of aromatic ether are represented by various peaks at 1745 cm^-1^, 1236 cm^-1^, and 1099 cm^-1^, respectively. These signature peaks confirm the presence of clove oil. Peaks in nanoemulsion CEO-NE spectra showed a broadening and blue shift on peak at 3467 cm^−1^ to3450 cm^−1^ associated with OH stretching. peak at 1638 cm^−1^ is associated with C = C in allyl group.^28^ In CEO-NE encapsulated CNPs all FTIR spectra show regions affiliated with the functional groups 1388 cm^−1^ (C–O–H, H–C–H), and 2857 cm^−1^ (C–H stretching). The appearance of the 1276 cm^−1^ (–P = O stretching) peak in the spectra indicates the TPP cross-linking in CEO-NE encapsulated CNPs.^45^ Peaks in CEO-NE encapsulated CNPs show the successful encapsulation of CEO-NE within chitosan nanocarriers as showed there was a broadening and red shift on peakat3467cm^−1^ to3487cm^−1^ associated with OH stretching attributed to the additional hydrogen bonds formed as a result of the existence of acetic acid, which used for chitosan nanoparticle synthesis.

The altered intensity and broadening of this peak along with the slight peak shift as observed in CEO-NE and CEO-NE encapsulated CNPs indicated the interaction between the individual components^27^. An intense peak was observed at 2930 cm^−1^ also seen in CEO (2925 cm^−1^) associated with OH stretching. It can be seen from the FTIR spectra that the addition of CEO-NE to CNPs led to a significant decrease in the intensity of OH stretching peak at 2925 cm^−1^, demonstrating an increment in the content of ester groups arising from CEO compounds. The changes in the wavenumbers of CEO-NE peaks were linked to the availability of CNPs, FTIR results indicated encapsulation of CEO-NE into chitosan NPs . Our results were in accordance with Ahmed Mahmoud Ismail etal (2024) ^46^.

### Effect of bio-agents on hatchability

The efficacy of clove and nano-clove emulsions as well combination of clove and chitosan were assessed as bio-agents against certain immature stages of *B. zonata* under laboratory conditions.

**Fig. 6:**
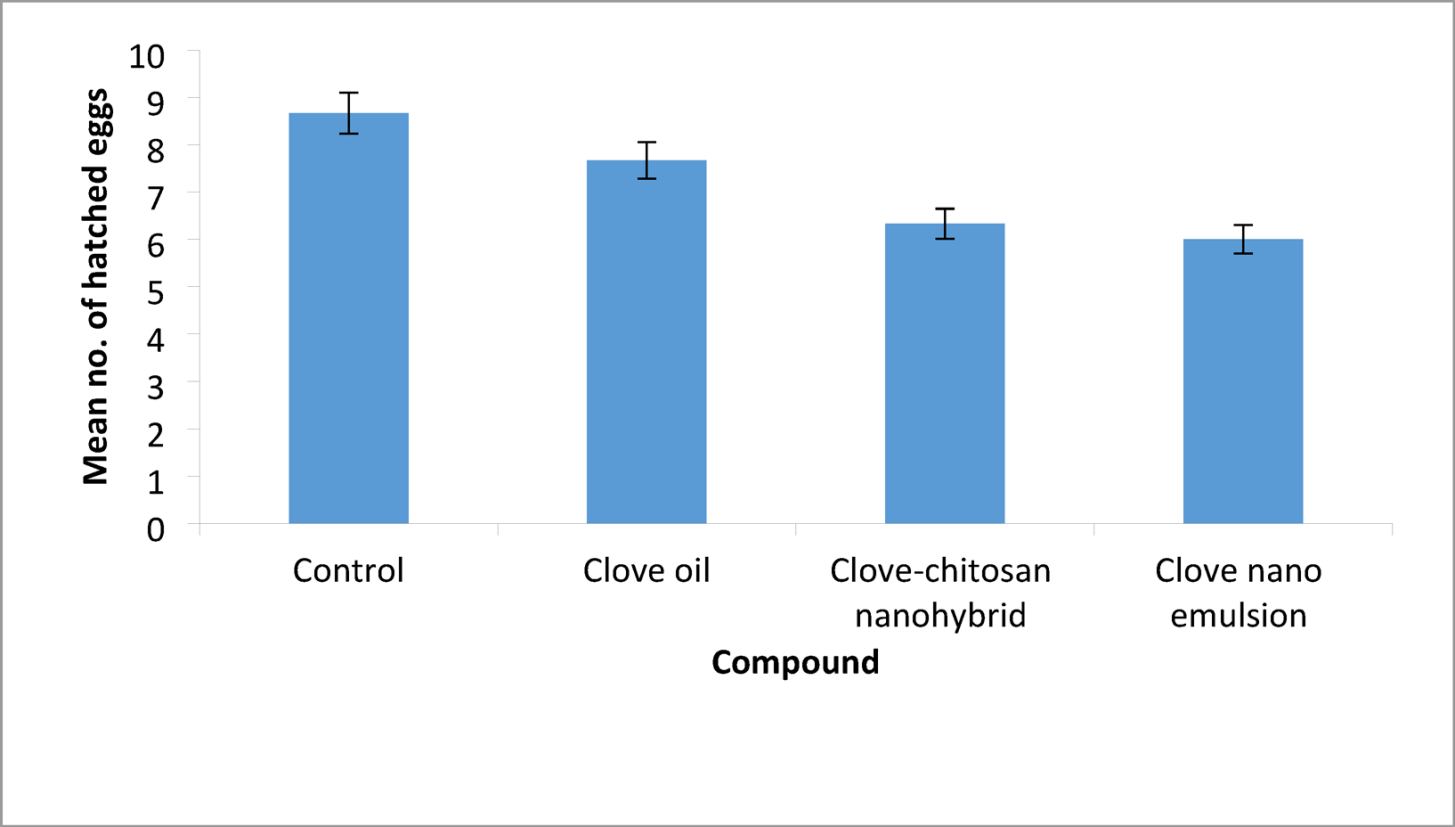
Effect of clove essential oils and emulsions of Clove nanoemulsion and Clove-Chitosan nanohybrid on hatchability of *B. zonata*, Averages followed by the same letter are not significantly different (*P* < 0.05).

Statistical analysis of data, compiled in Fig. 6**,** indicate that the differences in egg hatchability between the compounds used were insignificant. The average number of hatching eggs of *B. zonata* arranged in descending order as 8.67 ± 0.67, 7.67 ± 0.67, 6.33 ± 0.88and 6.00 ± 0.58eggs for the untreated individuals, clove, Clove-chitosan nanohybrid and Clove nanoemulsion, Emulsions of both nano-clove and Clove-chitosan nano hybrid showed equal efficiency recording the lowest hatching eggs of 6.00 ± 0.58and 6.33 ± 0.88, respectively. Also, the concentration used of the tested compounds insignificantly affected hatching eggs that ranged between 71.67- 6.00.

### Effect of bio-agents on larval mortality

**Fig. 7:**
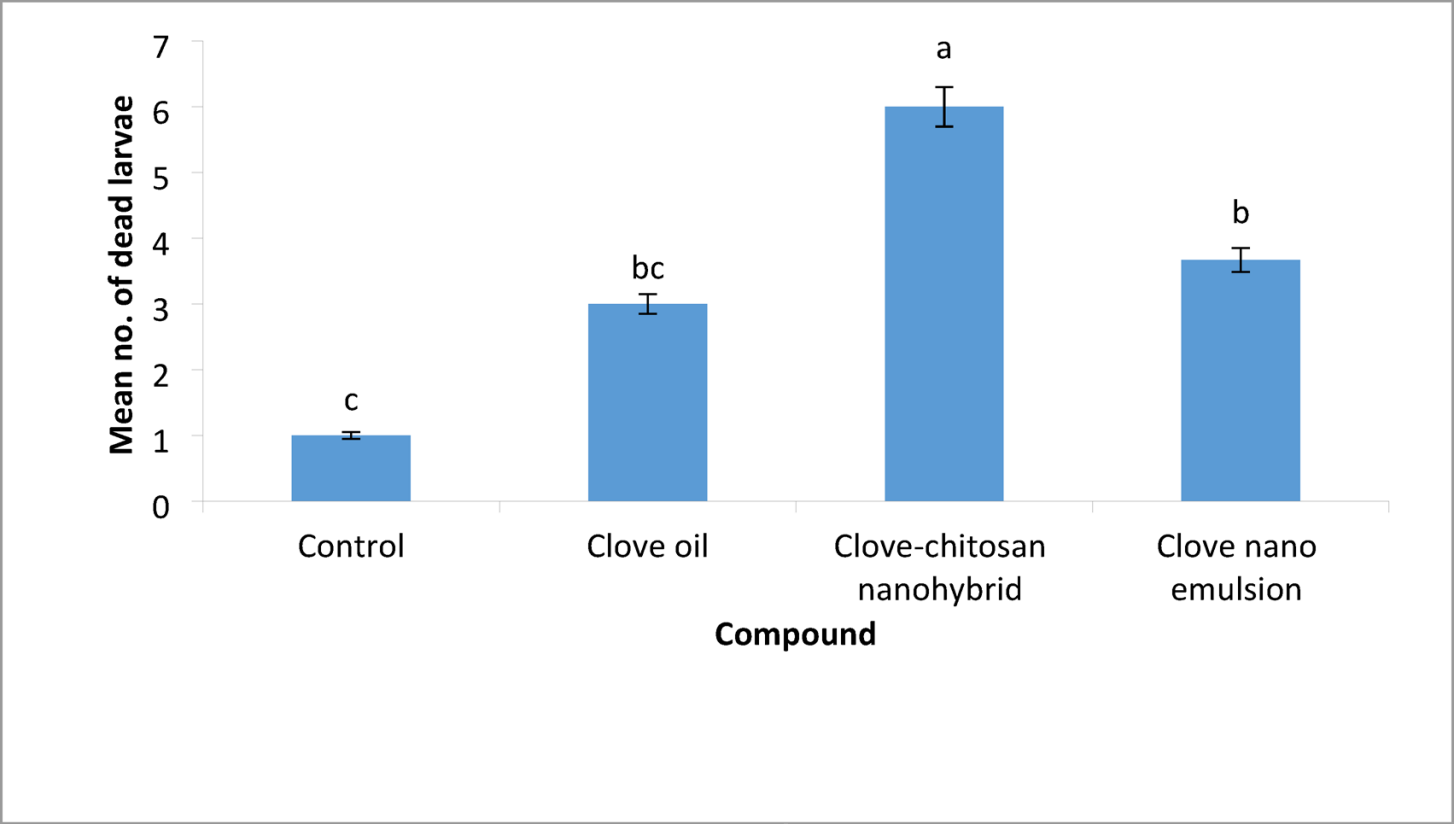
Larval mortality of *B. zonata* fed on treated artificial diet with clove essential oils and emulsions of Clove nanoemulsion and Clove-Chitosan nanohybrid, Averages followed by the same letter are not significantly different (*P* < 0.05).

Data in Fig. 7 indicates the average numbers of dead individuals throughout the three larval instars of the peach fruit fly fed on a treated larval artificial diet. The highest average number of dead larvae (6.00 ± 0.58, individuals) was significantly shown with Clove-Chitosan nanohybrid. The differences in dead larvae of both Clove Nano emulsion and clove only were statistically insignificant (3.67 ± 0.33 and 3.00 ± 1) which were significantly different compared to the untreated ones that exhibited the lowest larval mortality rate of 10%. Clove-Chitosan nanohybrid was the most effective showing the highest % larval mortality of 60%. This larval mortality could be caused by disruptive effects within the larvae’s body, such as a) gut epithelial layer fragmentation, microvilli gaps, and fat body degradation or disappearance. When these structures come into touch with eucalyptus oil, they are impacted and eventually die.^47^

### Effect of bio-agents on pupation

**Fig. 8:**
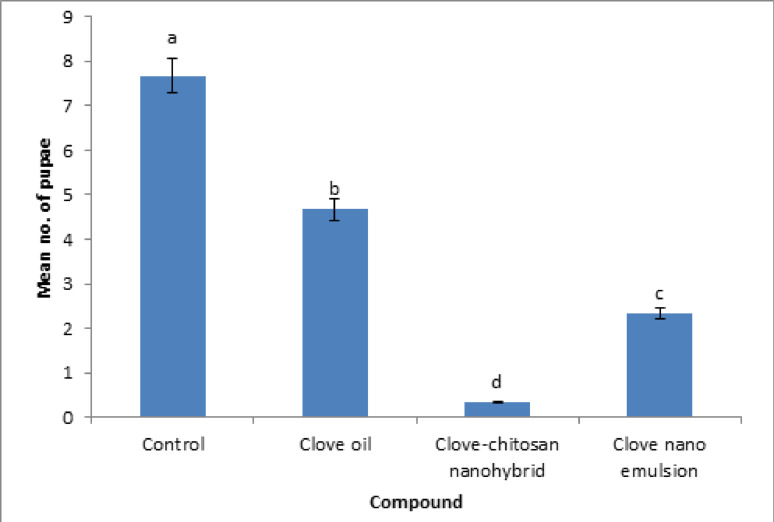
Effect of clove essential oils and emulsions of Clove nanoemulsion and Clove-Chitosan nanohybrid on pupation of *B. zonata*, Averages that are denoted by the same letter do not exhibit significant differences (P < 0.05).

As illustrated in Fig. 8, the statistical analysis of variance demonstrated that the differences in the number of formed pupae across treatments were highly significant. The highest % pupation (76.7%) was investigated with the untreated individuals that were significantly followed by clove essential oils of 46.7% pupation. Clove-Chitosan nanohybrid was the most potent, recording the lowest % pupation of 3.3% that is significantly followed by Clove nanoemulsion (23.3% pupation). The interference of the plant’s active metabolites with the bio-formation of the ecdysone hormone, which affects cytochrome P450 involved in the regulation of insect molting, may be the cause of the unsettling effects on insect development, such as prolonged larval duration and inhibition of molting.^48^

### Effect of bio-agents on normal adults

**Fig. 9:**
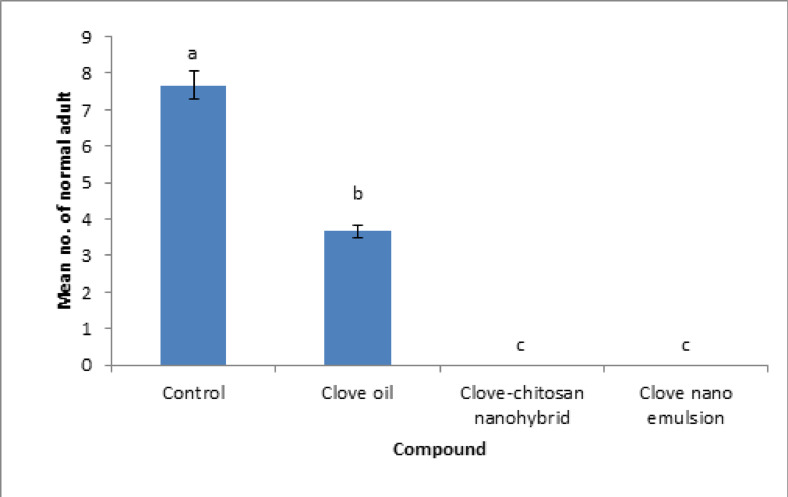
Effect of clove essential oils and emulsions of Clove nanoemulsion and Clove-Chitosan nanohybrid on normal adultnumbers of *B. zonata.* Averages followed by the same letter are not significantly different (P < 0.05).

Data shown in Fig. 9, reveal that emulsions of both Clove-Chitosan nanohybrid and clove nanoemulsion were the most efficient showing the lowest values of % emergence. The emerged adults investigated with emulsion of Clove-Chitosan nanohybrid was nil the same as those recorded with Clove nanoemulsion. Clove essential oils only showed high % emergence of 36.7 that significantly differed with those of the untreated ones (76.7% emergence).

### Effect of bio-agents on malformed adults

**Fig. 10:**
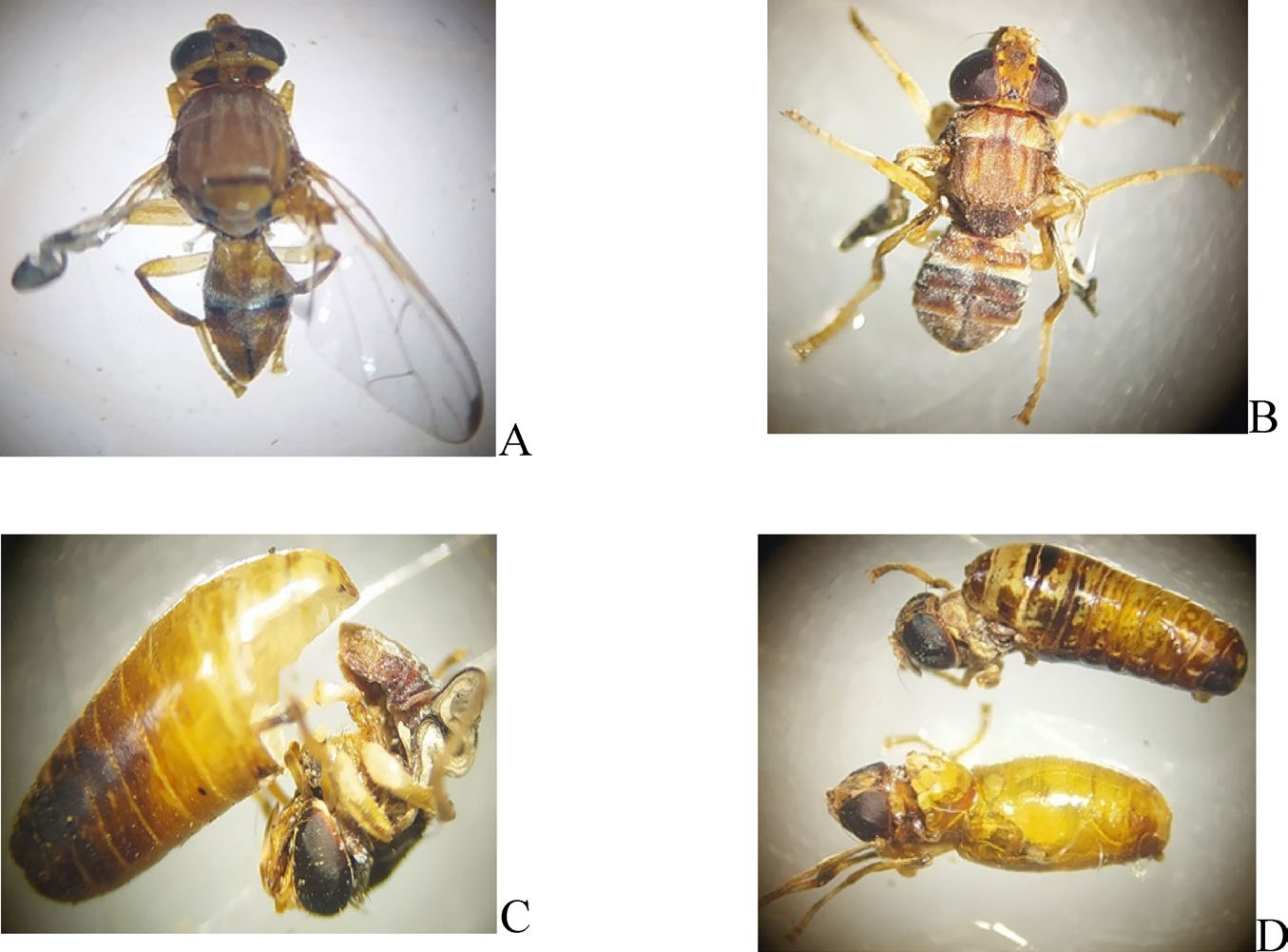
Several forms of deformed individuals of *B. zonata* resulted in treatments (**A**) Adult of a wing curled, (**B**) Adult with wing severely curled, (**C**) Adult attached with abdomen to puparium, (**D**) Adults emerged with head only.

**Fig. 11:**
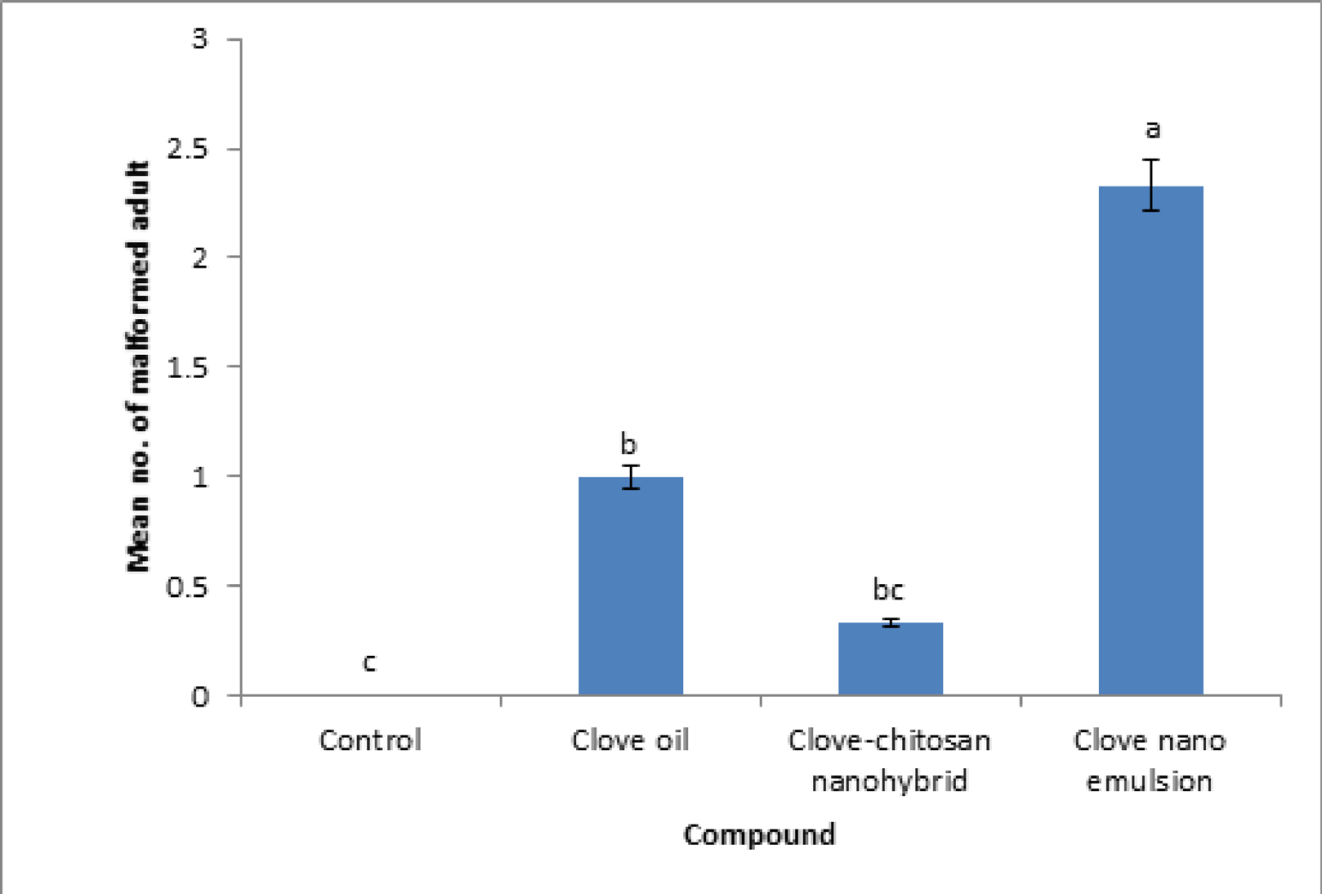
Malformed emerged adults of *B. zonata* recovered from larval artificial diet treated with Clove nanoemulsion and Clove-Chitosan nanohybrid.

Data in Figs. 10, 11 indicates that the tested compounds had teratogenic effects against *B. zonata*. The immature stages recovered from larval artificial diet treated with the tested compounds gave differently high percentages of malformed adults. Clove-Chitosan nanohybrid was the most potent compound suppressing pupal formation and adult emergence. Also, Clove nanoemulsion caused a high average number of deformed adults (2.33 ± 0.33 insects) that significantly differed with those recorded with clove only (1.00 ± 0 individuals). Abnormalities may result from the effects of certain compounds that inhibit metamorphosis, thereby disrupting hormonal regulation. El Kattan et al. (2011) observed a range of morphological abnormalities at all developmental stages of M. domestica when exposed to L. camara, *P. zonale*, C. macrocarpa, and A. nilotica. In a similar fashion, El Sherbini and Hany Kamel (2015) noted developmental irregularities in the larvae and pupae of M. domestica following treatment with *Fortunella crassifolia* extract, while Abdel Razik et al. (2017) reported significant malformations in larvae, pupae, and adults of *M. domestica* induced by administering certain plant extracts to 2nd instar larvae. Additionally, Abd El-Hamid et al. (2018) corroborated these findings when they treated samples with chemically synthesized AgNPs.^48^

### Effect of bio-agents on cumulative mortality

**Fig. 12:**
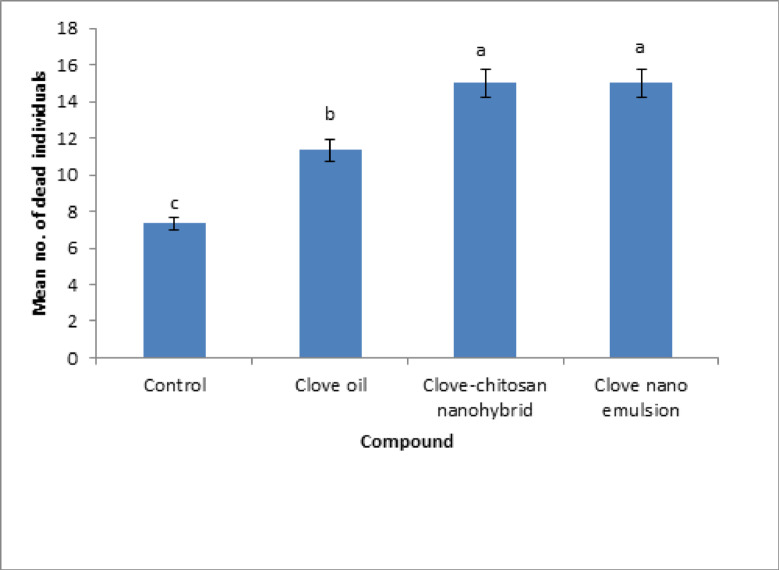
Cumulative mortality.

Data in Fig. 12, reveal that emulsions of both Clove-Chitosan nanohybrid and clove nanoemulsion were the most efficient showing the highest values of % cumulative mortality. Then the clove oil recorded 11.33 ± 0.33 of cumulative mortality and this result was significantly to the untreated individuals 7.33 ± 0.8.

Due to their low toxicity to humans, animals, the environment, and nontarget organisms, plant oils may present viable possibilities for use as pest control agents. They contain a variety of active, environmentally benign components with insecticidal characteristics. The insecticidal effects of clove oil are likely related to eugenol-induced neurotoxicity, interference with cuticle formation, and disruption of digestive and respiratory systems. Chitosan may enhance efficacy by altering insect gut permeability and acting as an antifeedant. The observed adult malformations, such as wing deformities and incomplete exclusion, are likely due to disruption of cuticle formation and interference with molting hormones caused by the clove nanoemulsion. ^49^

In contemporary times, EOs are utilized as biopesticides – pivotal for the successful commercial cultivation of plants, they are also regarded as Environmentally friendly.^22^ Nonetheless, planting essential oils are susceptible to deterioration by external influences such as air, light, moisture, and elevated temperatures. Consequently, nEOs were formulated to address the drawbacks of EOs using nanoemulsion technology. In this study, clove nanoemulsion was specifically developed. The findings were also consistent with prior research. Mustafa and Hussein et al. (2020) indicated that pesticide nanoemulsions possess significant potential for creating lipophilic active loaded products and advancing the development of nanoemulsion such as carriers or nano delivery systems for plant preservation. *Tang *et al*.* (2013) demonstrated that the efficacy of nanoemulsion could reach up to 60% against wild cabbage. Bian (2013) reported that the product exhibited enhanced insecticidal effectiveness of nanoemulsion against diamondback moths. *Shen *et al*.* (2012) revealed that nanoemulsion with an average size of 11 nm had a significant lethal effect on insects with minimal environmental contamination. Furthermore, *Chen *et al*.* (2012) showed that nanoemulsion with particle sizes of 10–100 nm provided an innovative agrochemical application system.^50^

Essential oils in the natural form or nano-emulsions as well as the natural product of chitosan were used for controlling several pests. Many researchers tried to assess the activity of the previous materials against several pests. The results obtained showed that nano forms of both clove oil and Clove-Chitosan nanohybrid had significant adverse biological effects starting from immature subsequently till adults of *Bactrocera zonata* throughout different paths as ingestion, contact and/or inhalation. These data are confirmed with those recorded by Ahmadi et al*.* reported that slow and persistent release of the essential oil of *Achillea millefolium*, proving the effectiveness of chitosan nanoencapsulation to prolong the acaricidal effect against adult of *Tetranychus urticae*.^51^ The lethality performance was influenced by the size of loaded chitosan nanoparticles. Also, nano formulation of this essential oil showed a long pesticidal effect. ^52^

Campos et al*.* observed decrement in toxicity of chitosan nanoparticles with carvacrol and linalool in nanoencapsulated form. The nanoparticles showed insecticidal activity against *Helicoverpa armigera* and *T. urticae* species. A reduction in mite egg laying was observed. ^53^ Essa et al. measured the effect of eucalyptus oil contained in nano encapsulated chitosan beads on *Culex pipiens* larvae, and the increase in mortality was proportional to the concentration recording larvicidal activity ^47^. Ferreira et al. observed the potentiality of chitosan nanoparticles of *Siparuna guianensis* essential oil in encapsulated form for the control of *Aedes aegypti* larvae ^54^. Efficacy against mosquito larvae was over 50% for 19 days with 100% mortality throughout the first week. El-Monairy *et al*. reported larvicidal activity of *Nerium oleander* leaf extracts and chitosan nanoparticles and silver synthesized against *M. domestica.*^55^

The tested compounds showed a decrease in the rate of pupation and adult emergence and caused significant deformations at various stages. Alakhdar and Gharib studied the relative toxicity of chitosan nanoparticles against four soybean pests in a semi-field experiment and showed that C.N.P was optimal against *Eutetranychus orientalis* after 14 days (74.36% mortality)^56^. Dhunnapa et al. evaluated the efficacy of nanoemulsions of lemongrass, clove, citronella grass, and cinnamon essential oils and their major compounds as toxicants and oviposition inhibitors against the African spider (*Eutetranychus africanus*) and revealed that EOs from citronella grass exhibited high toxic effects, causing 100% mortality at a concentration of only 0.6%, while geraniol (the major compound in citronella grass) caused the highest oviposition inhibition at a concentration of 0.2%.

Giunti et al*.* studied the residual contact toxicity and oviposition inhibition of three nanoemulsions based on the essential oils of *Foeniculum vulgare*, *Pimpinella anisum*, and *Mentha piperita* against *B. osmanthus* adult moths reported a reduction in the number of bites during egg laying in no-choice trials to all mint repellent formulations tested ^57^.

By verifying the efficacy of oil nanoemulsion as innovative delivery systems for utilizing the biopesticidal potential of plant oils, our study makes a substantial contribution to the field.

This supports earlier studies that found nano-formulations to have superior insecticidal activity over their bulk counterparts, demonstrating the technology’s exciting potential for creating long-term pest management solutions.^58,59^

The results and conclusions drawn from this research highlight the potential of utilizing nano-emulsion-based plant oils as the forthcoming generation of safe nano-pesticides. Consequently, additional research is required to evaluate their efficacy in actual field settings.

## Conclusion

In this study Clove nanoemulsion and Clove-Chitosan nanohybrid were prepared in this study using a simple and environmentally friendly method with high stability. As demonstrated in the discussion, the Clove-Chitosan nanohybrid is the most effective bioagent against B. zonata, recording the lowest percentage of egg hatchability and the highest percentage of larval mortality. Additionally, note the lowest percentage of pupation and display the lowest percentage of adult emergence. Therefore, clove oil’s effectiveness as an environmentally benign pesticide is increased when chitosan and clove are combined as a nanohybrid.

## Data Availability

The datasets used and/or analyzed during the current study are available from the corresponding author on reasonable request.
